# Supplementary material for: Valorizing Carasau Bread Residue Through Sourdough Fermentation: From Bread Waste to Bread Taste
Source: Microorganisms. 2025 Jul 25;13(8):1745. doi: 10.3390/microorganisms13081745 (PMC12388447; doi:10.3390/microorganisms13081745)
Supplement: Supplementary file 1 [file microorganisms-13-01745-s001.zip › microorganisms-3739370 Supplementary Figures.pdf]

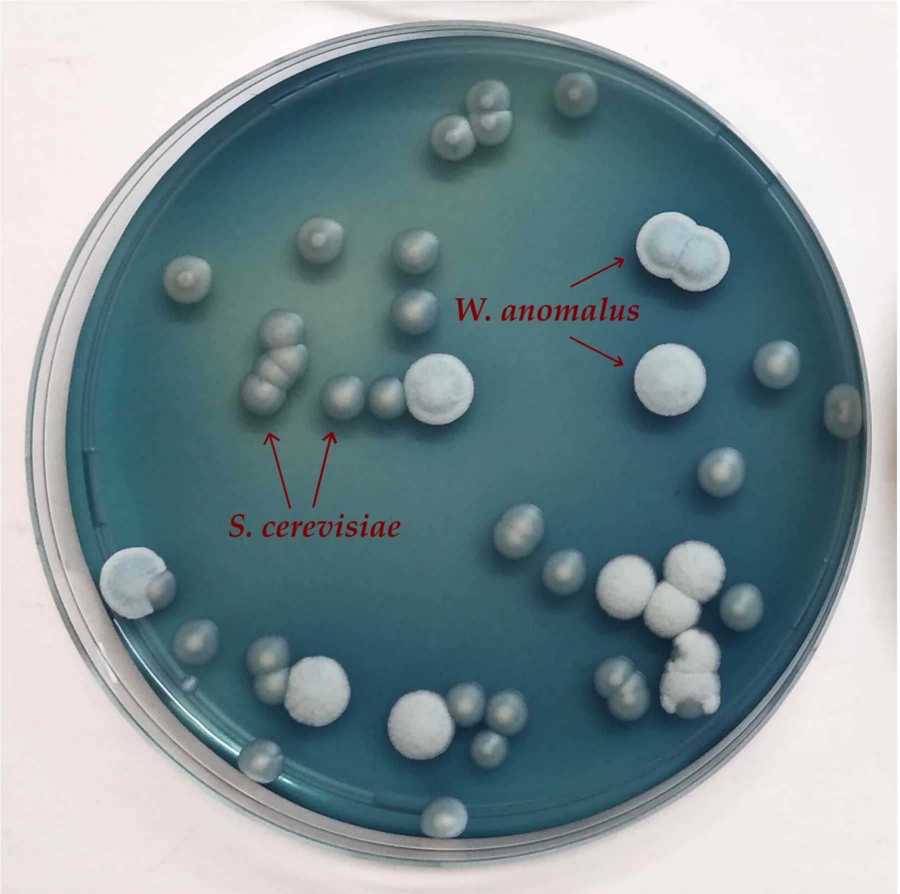

Figure S1. Differences in *S. cerevisiae* and *W. anomalus* colonies growing on W.L Nutrient agar medium.

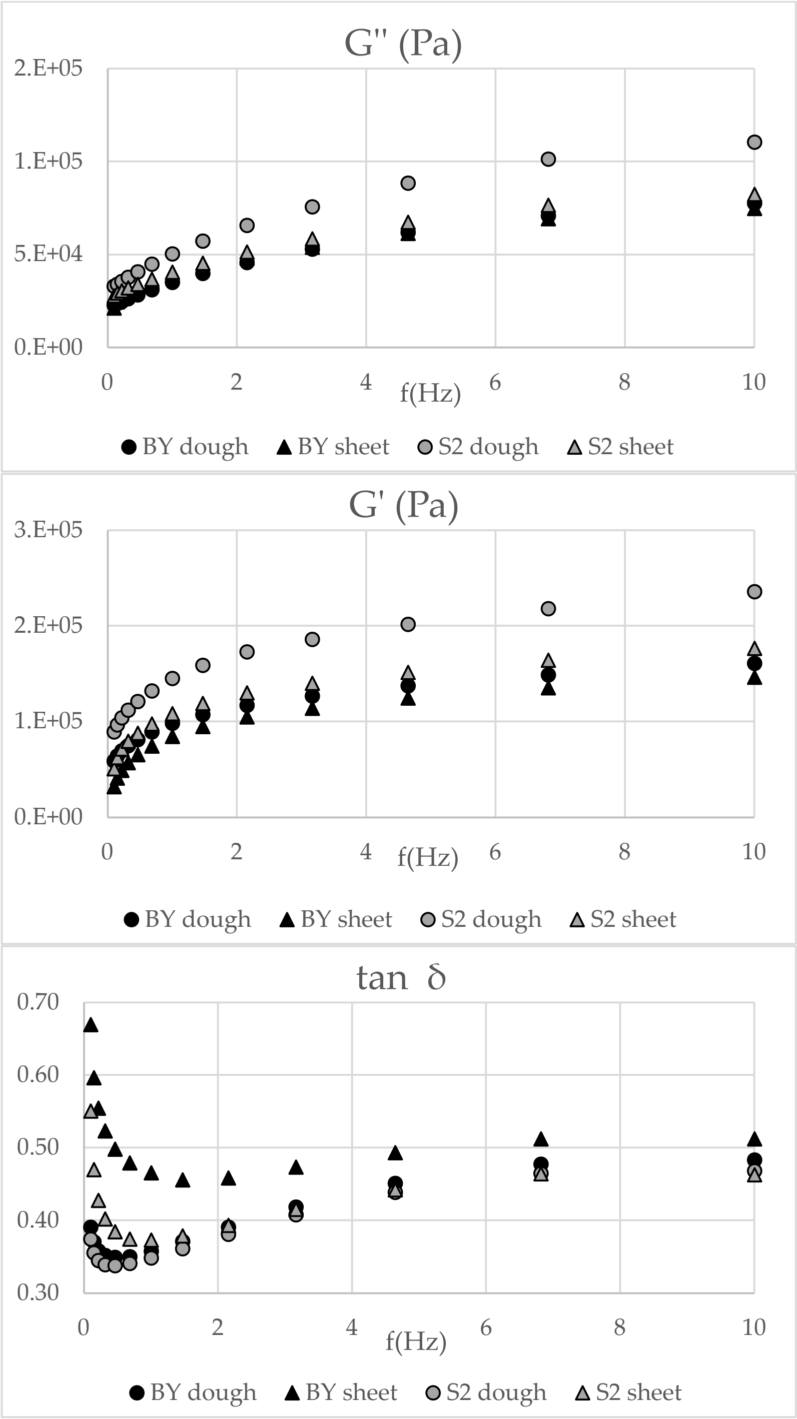

Figure S2. Storage modulus G′, loss modulus G″, and loss tangent tanδ as a function of frequency (Hz) in baker's yeast (BY) doughs and sheets, and in S2 doughs and sheets.
