# Supplementary material for: Valorizing Carasau Bread Residue Through Sourdough Fermentation: From Bread Waste to Bread Taste
Source: Microorganisms. 2025 Jul 25;13(8):1745. doi: 10.3390/microorganisms13081745 (PMC12388447; doi:10.3390/microorganisms13081745)
Supplement: Supplementary file 1 [file microorganisms-13-01745-s001.zip › microorganisms-3739370-supplementary tables.pdf]

Table S1. Sensory descriptors of Carasau bread (*n*=25) evaluated by CATA.

Sensory modality	Descriptors
Appearance	very toasted
	burnt areas
	bitter
	rustic
	uniform colour
	lightly toasted
	golden
Smell	semolina
	sourdough
	biscuit
	intense
	crumb
	bran
	baker's yeast
	abnormal
Taste	sweet
	slightly acidic
	stale
	tasty
Texture	brittle/ breaks easily
	very crispy
	sticks to the teeth
	very rough touch
	very thin
	very hard

Table S2. Properties of dough after mixing, leavening and baking of Carasau bread. Lowercase letters indicate significant differences ($p<0.05$). Standard deviations are reported

Samples	Parameters	Baker's yeast bread	Sourdough bread
Dough after mixing	Moisture (%)	41.32_a ±0.66	39.34_b ±1.28
	pH	6.3_a ±0.05	6.32_a ±0.09
	TTA	4.06_a ±0.4	3.81_b ±0.32
Leavened dough Sheets	Moisture (%)	37.18_a ±0.45	35.60_b ±0.38
	pH	6.21_a ±0.09	5.81_b ±0.39
	TTA	4.13_b ±0.24	5.22_a ±0.28
	Leaves weight (g)	199.6_a ±10.29	199.4±5.25 a
Carasau bread	Moisture (%)	4.88_b ±0.20	5.21_a ±0.26
	pH	6.25_a ±0.15	5.69_b ±0.29
	TTA	4.64_b ±0.13	7.26_a ±0.40

Table S3. Frequency values of the sensory attributes of the CATA analysed with the Cocran test. Lowercase letters indicate the significant differences.

Attributes	Baker's yeast bread	Sourdough bread	p-value
very toasted	8_a	57_b	<0,0001
scorched areas	12_a	82_b	<0,0001
tasty	54_a	87_b	<0,0001
golden tint	78_a	92_b	0.035
uniform color	86_b	39_a	<0,0001
very crunchy	66_a	102_b	<0,0001
sourdough smell	4_a	13_b	0.029
biscuit smell	8_a	20_b	0.014
bran smell	4_a	22_b	0.000
very rough to the touch	56_a	83_b	0.001
rustic appearance	44_a	100_b	<0,0001
